# A new method for axis adjustment of the hydro-generator unit using machine learning

**DOI:** 10.1038/s41598-023-30121-0

**Published:** 2023-02-20

**Authors:** Jie Cao, Yang Li, Zhaoyang Qu, Yunchang Dong, Yaowei Liu, Ruxuan Zhang

**Affiliations:** 1 School of Computer Science, Northeast Electric Power University, Jilin, China; 2 Guangdong ATV Academy for Performing Arts, Dongguan, China; 3 School of Electrical Engineering, Northeast Electric Power University, Jilin, China; 4 State Grid Jilin Electric Power Co., Ltd., Changchun, China

**Keywords:** Electrical and electronic engineering, Energy infrastructure, Mechanical engineering, Computer science

## Abstract

The power quality and efficiency of the hydro-power station depend on the stable operation of the hydro-generator unit, which needs to continue to operate and it is prone to axis failure. Therefore, to adopt effective axis adjustment technology to eliminate faults. This paper proposes a new method for axis adjustment of hydro-generator unit based on an improved grey prediction model and swarms intelligence optimization neural network. First of all, it proposes a sequence acceleration translation and mean value transformation method, which is used to pre-process the axis net total swing sequence that exhibits oscillating fluctuations. It uses *e*_1_ and *e*_2_ factor transformation to establish an improved axis net total swing gray prediction model. Then, the advanced flamingo search algorithm is used to search the maximum value of the sine function of the net total pendulum of the axis, and the axis adjustment orientation is obtained. This method solves the problem that GM(1, 1) can only be predicted by monotone sequence in the past and the problem that the search algorithm is easy to fall into local optimum, effectively improves the calculation efficiency of axis and shorts the search time. Simulation examples show that the proposed method can significantly improve accuracy of axis adjustment. This method greatly improves the efficiency of azimuth search for axis adjustment.

## Introduction

Hydro-generator unit (HGU) is a part of the key equipment of hydro-power station^1,2^. State of the reliable measurement of the trend of hydroelectric generating set axis for unit security, it promotes the power system stability of great significance^3^. In practical engineering, axis adjustment is the most important work in the later period of unit installation. Overhaul of the unit must also go through axis adjustment inspection. The quality of the unit axis comprehensively reflects the quality of installation and maintenance^4^. Axis adjustment central axis of the net total swing and maximum net total swing degrees is an important parameter of axis adjustment calculation, so the degree of the axis of the hydroelectric generating set net total swing and prediction as well as to the axis of maximum net total swing adjustment bearing search, to guarantee the safety of the hydro-generator unit and reduce economic loss of the hydro-power station is of great significance^5^.The calculation of net total swing and relative swing in axis adjustment of the hydro-generator unit is still mainly by manual calculation. The Axis calculation process is complicated, it is involving the measurement data of upper lead, lower lead, water lead, and other parts, which need the dimensions of upper lead to lower lead, upper lead to water lead, thrust head diameter, and so on. Therefore, the efficient and accurate prediction method of axis net total swing is conducive to reducing the number of axis measurements, and it is also the basis and prerequisite for realizing axis measurement^6^. There are four typical prediction algorithms for the prediction of net total swing and maximum net total swing of hydro-generator axis in machine learning methods: the prediction method using BP neural network (BP); Prediction method using support vector machine (SVM); physics-informed neural network (PINN); Prediction method using grey model (GM). There are two main methods of axis adjustment azimuth search: traditional manual processing and swarm intelligence algorithm. At present, machine learning has been widely used in many fields, but not many applications have been applied to the axis adjustment of the hydro-generator unit^7^. However, for axis adjustment orientation, machine learning can solve the problem that axis adjustment orientation is difficult to search by using algorithms^8,9^.

To solve the above problems, this paper proposes a new method for axis adjustment of the hydro-generator unit using machine learning. Its main contributions include:

1. A prediction method of axis net total swing using the improved GM(1, 1) is proposed. Through accelerating translation and meaning transformation methods, the net total swing sequence shows oscillatory fluctuations are pre-processed to weaken the series volatility. This model solves the problem that GM(1, 1) can only be predicted by monotone sequence. Then, the improved GM(1, 1) algorithm can be used to predict axis net total swing data and improve the axis measurement processing efficiency.
2. An optimization method of axis adjustment orientation search using flamingo search algorithm is proposed to accurately search axis adjustment orientation. This model solves the problem that traditional search algorithm is easy to fall into local optimal solution.
3. This paper proposed method with the traditional manual axis processing, and compare it with the representative machine learning algorithm. The performance evaluation shows that can significantly improve the search of axis adjustment orientation by the model.

In the area of axial net total swing prediction, a classifier combining rough sets and the support vector machine was proposed in the literature^10,11^, it was applied to fault diagnosis of HGU. An HPG vibration trend measurement model was proposed in the literature^12^, which is based on optimal variational mode decomposition (OVMD) and chaotic sine–cosine algorithm optimization (CSCA) and improved least squares support vector machine (LSSVM). The literature^13^ proposed a Guassion regression process (GPR) based early fault prediction method for HPG, which effectively tracked the change process of HPG operation status and monitors the abnormal operation status of HPGs in advance. Literature^14^ proposed a least squares support vector machine (LS-SVM) method for predicting the stability parameters of mixed-flow hydro turbine units. The literature^15^ developed a time series prediction model ARIMAX (1, 1, 1) for hydroelectric power generation in ecuador to predict the monthly output for up to one year. The literature^16–18^ proposed a grey prediction model (GM(1, 1)) for predicting the performance degradation trend of hydroelectric units. The literature^19,20^ proposed a meta-learning technique for offline discovery of physics-informed neural network (PINN) loss functions. It extend earlier works on meta-learning, and develop a gradient-based meta-learning algorithm for addressing diverse task distributions based on parametrized partial differential equations (PDEs) that are solved with PINNs. Although the above methods improved the efficiency of the axial measurement under certain conditions, the selection of the neural network needs to consider the size of the data set as well as the setting of the parameters, so there are inevitable errors. In addition, they are not all suitable for the data set of net full swing of the axis of the hydro-generator set.

In terms of axial adjustment orientation search, literature^21,22^ proposed an improved adaptation evaluation method, it put forward the concept of stability and instability parameter sets, and it applied to the stability search strategy, which effectively improved the local search capability of the model algorithm and it ensured the stability and accuracy of the hydro-generator set model. The literature^23–26^ proposed a prediction method based on radial basis function (RBF) interpolation, empirical modal decomposition (EMD), approximate entropy, artificial neural network, and grey theory for the degradation of hydro-generator unit characteristic parameters. A nonlinear prediction model for the degradation trend of hydro generator unit operating parameters was proposed in the literature^27–31^. The model was based on radial basis function interpolation, wavelet transform, maximum lyapunov exponent prediction method, and gray prediction model (GM(1, 1) method). The literature^32^ put forward the dynamic alarm curve model, established GA-BP neural network model, and it obtained the vertical vibration of head covering by training and outputting the alarm dynamic curve. Although the above methods can predict the axis adjustment amount at a certain moment, most of them belong to single-step prediction, and when it is necessary to know the changing trend of the axis adjustment amount of the hydro-generator unit earlier, the single-step prediction model needs to be extended to a multi-step prediction using rolling prediction^33–35^, which time the prediction accuracy will be reduced due to the continuous superposition of errors each time, which cannot meet the actual need of hydro-generator set maintenance project. The machine learning algorithm is an important method to solve prediction and optimization. Based on the improved grey model, the data series with oscillating wave phenomena are pre-processed by accelerating translation and meaning transformation method to weaken the series volatility^36–38^, and then the prediction function is obtained by factor transformation. The flamingo algorithm is a new swarm intelligence optimization algorithm based on the migration and foraging behavior of flamingos^39–41^. It can increase the ability of searching and develop search space, ensure a good balance between search and development, and it can effectively solve nonlinear optimization problems. The improved RBF neural network uses particle swarm optimization to determine the center of the RBF neural network^42–44^, it controls the optimization speed by the inertia weight factor, and it takes the maximum net total swing of the maintenance data of the hydro-generator unit as the input vector of the neural network to predict the real axis adjustment amount^45–47^.

## Methods

In this paper, the net total swing at the axis adjustment point is used as the state quantity to represent the operating state of the hydro-generator unit. The improved grey model is used to predict the net total swing at the axis adjustment point in the future, and the predicted values of the net total swing of the axis at eight state points around the big axis are obtained respectively. Next, the sine function fitting of the net total swing value of the eight-point axis is carried out to obtain the sine function, and then, on the basis of obtaining the sine function of the net total swing. Then, using the obtained net total swing sine function, the flamingo search algorithm is used to search the maximum value of the sine function of net total swing at the axis adjustment, and the corresponding axis adjustment orientation is obtained.

**Figure 1:**
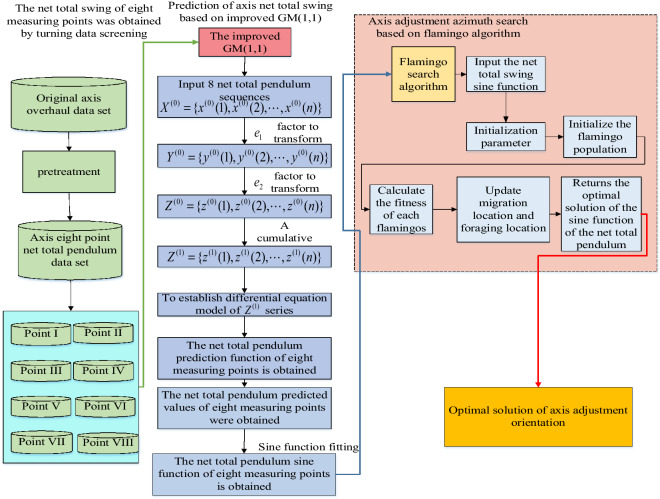
Framework diagram of the axial alignment model.

The determination of axis adjustment orientation and axis adjustment amount is also realized. The general framework is shown in Fig. 1.

In this section, we review the relevant data of the hydro-generator unit and observing the axis maintenance data set, it is found that the axis net total swing sequence shows a fluctuation phenomenon. To solve the problem that the axis net total swing has oscillation fluctuation, this paper uses accelerated translation and weighted mean transformation to preprocess the axis net total pendulum with fluctuation phenomenon, and then improved GM(1, 1) modeling after meeting the monotone condition.

### Net total swing prediction based on improved GM(1, 1) model

#### GM(1, 1) model

The basic idea of the traditional GM(1, 1) model: to facilitate mathematical modeling, the original net total swing sequence is accumulated once. Since the sequence after accumulation has an exponential growth trend, the approximate first-order differential equation is used to build the model. Finally, the prediction sequence is generated by the accumulation of the modeling sequence to complete the prediction of the development trend of the original net total swing sequence.

The specific modeling process for the traditional GM(1, 1) is as follows.

Let the original net total swing sequence be: $X^{{_{(0)} }} = \{ x^{(0)} (1),x^{(0)} (2), \ldots ,x^{(0)} (n)\}$, and it performs a single accumulation on this sequence to generate. As Eq. (1).

1 $$X^{{_{(1)} }} (k) = \sum\limits_{i = 1}^{k} {x^{(0)} (i),K = 1,2, \ldots ,n}$$

Generate a net total swing sequence with exponential regularity as $X^{(1)} = \{ x^{(1)} (1),x^{(1)} (2), \ldots ,x^{(1)} (n)\}$.

$X^{(1)}$ approximates the sequence as a first-order differential (Eq. 2) equation’s solution.

2 $$\frac{{{\text{d}}x^{(1)} }}{dt} + ax^{(1)} = b$$

where: *a* is the development coefficient of the model; *b* is the amount of grey action.

Denote the parameter $A = [a,b]^{T}$, and it uses the least-squares method to find *A* as

3 $$A = (B^{T} B)^{ - 1} B^{T} Y$$

where:

$$B = \left[ {\begin{array}{*{20}c} {\begin{array}{*{20}c} { - \frac{1}{2}[x^{(1)} (1) + x^{(1)} (2)]} & 1 \\ \end{array} } \\ {\begin{array}{*{20}c} { - \frac{1}{2}[x^{(1)} (2) + x^{(1)} (3)]} & 1 \\ \end{array} } \\ {\begin{array}{*{20}c} \vdots & \vdots \\ \end{array} } \\ {\begin{array}{*{20}c} { - \frac{1}{2}[x^{(1)} (n - 1) + x^{(1)} (n)]} & 1 \\ \end{array} } \\ \end{array} } \right]$$

$$Y = \{ x^{(0)} (2),x^{(0)} (3), \ldots ,x^{(0)} (n)\}^{T}$$

Find the values of *a*, *b* and substitute them into Eq. (2) to calculate.

4 $$\mathop x\limits^{ \,\,\, \, \, \wedge(1)} (k + 1) = [x^{(0)} (1) - \frac{b}{a}]e^{ - ak} + \frac{b}{a}$$

The cumulative reduction from Eq. (4) yields the net total swing prediction function as

5 $$\mathop x\limits^{\,\,\, \, \, \wedge(0)} (k + 1) = \mathop x\limits^{\,\,\, \, \, \wedge(1)} (k + 1) - \mathop x\limits^{\,\,\, \, \, \wedge(1)} (k) = (1 - e^{a} )[x^{(0)} (1) - \frac{b}{a}]e^{ - ak}$$

In the traditional GM(1, 1) modeling, because the net total swing of the original axis sequence is not very regular, it is necessary to carry out cumulative changes and then it uses Eq. (2) to build a mathematical model. This modeling method regardless of whether the axis net total swing original sequence oscillates or not, the cumulatively generated sequence will change monotonically, and the reduced sequence also shows the same change trend. When the net total swing of the original sequence is monotonic, the prediction accuracy is better, but when the original sequence oscillates and fluctuates, the prediction accuracy is not ideal because the reduced sequence changes monotonically and the original swing sequence cannot be fitted accurately.

If the axis net total swing oscillation wave sequence is transformed mathematically to make it have a monotone trend, the mathematical model is established by GM(1, 1), finally, the reduction function is calculated, and then the mathematical inverse transformation is carried out to obtain the axis net total swing prediction sequence, it will be a good solution to the problem that the traditional GM(1, 1) model does not have high prediction accuracy for the oscillating sequence. In this paper, the accelerated translation and meaning transformation method are combined to pre-process the series with oscillatory fluctuation phenomenon, to weaken the fluctuation of the series.

$X = \{ x(1),x(2), \ldots ,x(n)\}$ is the axis net total swing original sequence, and if there exists $k,k \in [1,2, \ldots ,n - 1]$, such that $x(k + 1) - x(k) > 0,x(k + 1) - x(k) < 0$, then it is said to $X$ be a sequence of random swings. As Eq. (6) and (7).

6 $$M = \max \{ x(k)|k = 1,2, \ldots ,n\}$$

7 $$m = \min \{ x(k)|k = 1,2, \ldots ,n\}$$

*M*–*m* is the amplitude of the sequence *X*, denoted as *T*.

Define accelerated translation transformation: in order to weaken the volatility of the axis net total swing of the original oscillatory sequence. $XE_{1} = \{ x(1)e_{1} ,x(2)e_{1} , \ldots ,x(n)e_{1} \}$, equation:

8 $$x(k)e_{1} = x(k) + (k - 1)T,k = 1,2, \ldots ,n$$

It is called *e*_1_ the accelerated translation transformation factor, and the monotonic trend of the original swing sequence after this transformation can be proved by simple mathematical operations.

Define the weighted mean transformation: the axis net total swing sequence after *e*_1_ factor processing has shown a monotonic trend and it has modeling conditions. To fit the axis net total swing of the original sequence more accurately, the weighted mean transformation is introduced to perform a quadratic transformation on the factorized sequence, and the transformed sequence is defined.

$$XE_{2} = \{ x(1)e_{1} e_{2} ,x(2)e_{1} e_{2} , \ldots ,x(n)e_{1} e_{2} \} ,\quad {\text{equation}}:$$

9 $$x(k)e_{1} e_{2} = (\sum\limits_{i = 1}^{k} {x(i)e_{1} } )/k$$

It is called *e*_2_ the weighted mean-transformed factor. Through simple mathematical calculation, it can be proved that the axis net total swing sequence after *e*_2_ factor transformation keeps the monotone characteristics of the axis net total swing original sequence, which can make the axis net total swing sequence smoother.

#### Improved GM(1, 1) modeling of oscillatory sequences

Let the axis net total swing of the original swing sequence be

$$X^{(0)} = \{ x^{(0)} (1),x^{(0)} (2), \ldots ,x^{(0)} (n)\} \quad x^{(0)} (k) > 0,k = 1,2, \ldots ,n$$

The improved GM(1, 1) modeling processes are as follows.

1. $X^{(0)}$ Perform an *e*_1_ factor transformation on the axis net total swing of original sequence as $Y^{(0)} = \{ y^{(0)} (1),y^{(0)} (2), \ldots ,y^{(0)} (n)\}$.
2. The sequence $Y^{(0)}$ is *e*_2_ factor-transformed to
  $$Z^{(0)} = \{ z^{(0)} (1),z^{(0)} (2), \ldots ,z^{(0)} (n)\}$$
3. Perform a summation of the sequence $Z^{(0)}$.
  $$Z^{(1)} = \{ z^{(1)} (1),z^{(1)} (2), \ldots ,z^{(1)} (n)\}$$
4. Modeling $Z^{(1)}$’s GM(1, 1) differential equation for the sequence. As Eq. (10).
  10 $$\frac{{dz^{(1)} }}{dt} + az^{(1)} = b$$
  *a*,* b* parameters by least squares. As Eq. (11).
  11 $$\left[ {\begin{array}{*{20}c} a \\ b \\ \end{array} } \right] = (B^{T} B)^{ - 1} B^{T} P$$
  It is determined, among other things.
  $$B = \left[ {\begin{array}{*{20}c} { - \frac{1}{2}[z^{(1)} (1) + z^{(1)} (2)]} & 1 \\ { - \frac{1}{2}[z^{(1)} (2) + z^{(1)} (3)]} & 1 \\ \vdots & \vdots \\ { - \frac{1}{2}[z^{(1)} (n - 1) + z^{(1)} (n)]} & 1 \\ \end{array} } \right]$$
  $$P = [z^{(0)} (2),z^{(0)} (3), \ldots ,z^{(0)} (n)]^{T}$$
5. The response function of the GM(1, 1) differential equation.
  12 $$\mathop z\limits^{\,\,\, \, \, \wedge(1)} (k + 1) = [z^{(0)} (1) - \frac{b}{a}]e^{ - ak} + \frac{b}{a}$$
6. The axis net total swing prediction function is obtained by one cumulative reduction $z^{(0)}$.
  13 $$\mathop z\limits^{\,\,\, \, \, \wedge(0)} (k + 1) = \mathop z\limits^{\,\,\, \, \, \wedge(1)} (k + 1) - \mathop z\limits^{\,\,\, \, \, \wedge(1)} (k) = (1 - e^{a} )[z^{(0)} (1) - \frac{b}{a}]e^{ - ak}$$
  Which: $$k = 1,2, \ldots ,n;\mathop z\limits^{\,\,\,\,\,\wedge (0)} (1) = z^{(0)} (1)$$.
7. For $Z^{(0)}$ use $e_{2}$ factor inverses transformation reduction to attain. As Eq. (14).
  14 $$\mathop y\limits^{\,\,\, \, \, \wedge(0)} (k) = k\mathop z\limits^{\,\,\, \, \, \wedge(0)} (k) - \sum\limits_{i = 1}^{k - 1} {\mathop y\limits^{\,\,\, \, \, \wedge(0)} (i),k = 2,3, \ldots ,n}$$
  where $$\mathop y\limits^{\,\,\, \, \, \wedge(0)} (1) = y^{(0)} (1)$$, and
  15 $$\mathop y\limits^{\,\,\, \, \, \wedge(0)} (k + 1) = (k + 1)\mathop z\limits^{\,\,\, \, \, \wedge(0)} (k + 1) - k\mathop z\limits^{\,\,\, \, \, \wedge(0)} (k)$$
8. The inverse transformation of the axis net total swing of the original sequence by $e_{1}$ factor pair $Y^{(0)}$ yields a prediction function. As Eq. (16).
  16 $$\mathop x\limits^{\,\,\, \, \, \wedge(0)} (k + 1) = \mathop y\limits^{\,\,\, \, \, \wedge(0)} (k + 1) - kT = (k + 1)\mathop z\limits^{\,\,\, \, \, \wedge(0)} (k + 1) - k\mathop z\limits^{\,\,\, \, \, \wedge(0)} (k) - kT$$
  Which: $$k = 1,2, \ldots ,n;\mathop x\limits^{\,\,\, \, \, \wedge(0)} (1) = x^{(0)} (1)$$.

**Figure 2:**
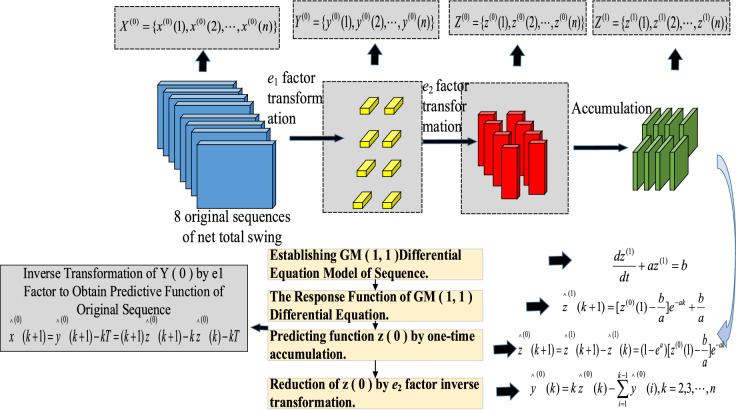
Framework diagram of the improved GM algorithm.

The above improved steps of GM(1, 1) and the formula after factor transformation are shown in Fig. 2.

### Adjusted orientation finding model based on the flamingo search algorithm

Obtained by the improved GM(1, 1) to predict the axis of the net total degree of the pendulum, the next it needs to be clean all degrees of sine function fitting and establish a large shaft bearing as the abscissa, net total swing as the ordinate axis net total swing degrees after sine fitting function, using the flamingo algorithm to the maximum net total swing of sine function optimization. Finally, the independent variable corresponding to the maximum value of sine function of axis net total pendulum is the axis adjustment azimuth.

Flamingo has the global search and local development capabilities needed for optimization algorithms. It has good results for single-peak and multi-peak function search. Therefore, it is suitable for searching the maximum value of the sine function of net total swing.

The two main behavioral traits of flamingos are foraging and migratory behavior. Flamingos primarily inhabit areas where food is plentiful. After a period of extensive foraging, the flamingo population migrates when the food in the area decreases to a level that does not satisfy the population. Corresponding foraging and migration models are developed.

The main optimization ideas of the FSA model are as follows.

### Foraging behavior

1. Communicative behavior
  Flamingos with the most food in the group spread their position information by calling other flamingos, and it influences the position changes of other flamingos in the group. Theoretically, flamingos cannot know where the most food is in an area. However, this does not mean that the algorithm cannot find a globally optimal solution, because the algorithm is a program, you cannot tell the end condition of the program when you set it.
  FSA is an algorithm that simulates flamingos searching for the best solution in a search area based on the limited available information. In this paper, we assume that the flamingo with the largest amount of food in the *j*th dimension is *xb*_*j*_.
2. Beak scanning behavior
  When a flamingo's beak is poured over water, it acts like a large sieve, and it is sucking the water and then quickly filtering it out, a foraging pattern influenced by the abundance of food in the area. If there is more food in the area swept by the flamingo's bill, this encourages the flamingo to scan the area more carefully and the flamingo's neck slowly stretches out, it is causing the beak's scan radius to increase. The likelihood of scanning the area for food will also increase, and a model of the flamingo's beak scanning behavior is shown in Fig. 3.

**Figure 3:**
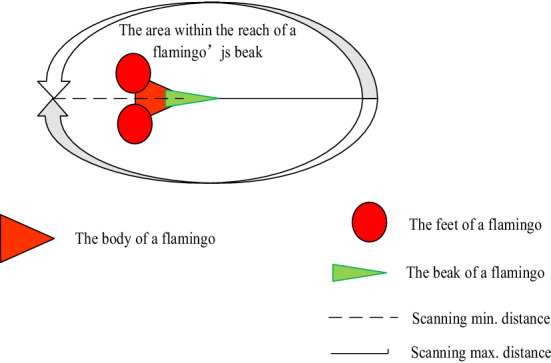
Beak scanning behavior.
  The closer a flamingo is to the most abundant food in the population, the greater the likelihood that food is abundant in that area. In this paper, we simulate the beak-scanning behavior of flamingos. Let the position of the *i*th flamingo on the *j*th dimension of the flamingo population is *x*_*ij*_, it is necessary to consider the variability of flamingo individual selection in nature and the suddenness of specific environment, which will affect the foraging behavior of flamingo. Otherwise, there are errors in the foraging behavior and information transmitted by flamingos. To model this error, a standard normal random distribution is introduced, in which the flamingo beak scan has a high probability of aligning with the position where the food is most abundant. However, there is also a small probability of error based on this information.
  The maximum distance of flamingo beak scans in foraging behavior can be quantified as $|G_{1} \times xb_{j} + \varepsilon_{2} \times x_{ij} |$, where $\varepsilon_{2}$ is a random number of − 1 or 1. The maximum distance is primarily intended to increase the search range of the flamingo beak scan in foraging mode, where *G*_1_ is a random number that fits a standard normal distribution. To model the scanning range of the flamingo in beak scanning behavior, the normal distribution is again introduced, and its variation curve approximates the variation of the flamingo's beak scanning range as $G_{2} \times |G_{1} \times xb_{j} + \varepsilon_{2} \times x_{ij} |$, where *G*_2_ is a random number obeying a normal distribution.
3. Bipedal mobile behavior
  A model of the foot movement behavior of flamingos is shown in Fig. 4. When flamingos forage for food, as they scan with their beaks, their talons move towards the most abundant food in the flamingo population. The distance traveled can be quantified by assuming that the most abundant food place in the population is $\varepsilon_{1} \times xb_{j}$ $xb_{j}$, $\varepsilon_{1}$ as a random number of − 1 or 1, it mainly to increase the search range of flamingos foraging and to quantify individual selection differences.

**Figure 4:**
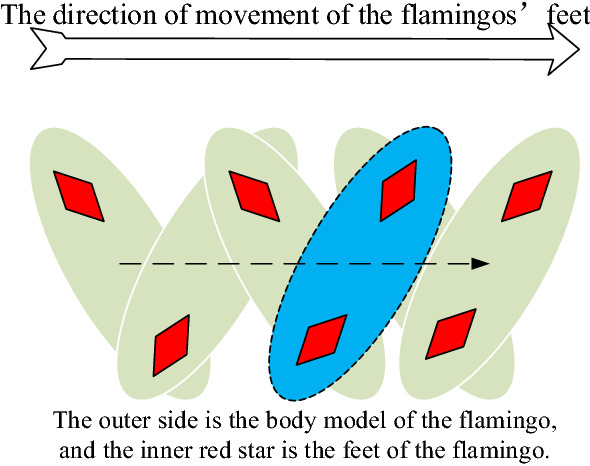
Bipedal mobile behavior.

In summary, the moving step of the flamingo foraging in the *t* iteration is the scanning range of the flamingo beak, and then the moving distance of its feet is added, as shown in Eq. (17).

17 $$b_{ij}^{t} = \varepsilon 1 \times xb_{j}^{t} + G_{2} \times |G_{1} \times xb_{j}^{t} + \varepsilon 2 \times x_{ij}^{t} |$$

The equation to update the location of flamingo foraging behavior is

18 $$x_{ij}^{t + 1} = (x_{ij}^{t} + \varepsilon_{1} \times xb_{j}^{t} + G_{2} \times |G_{1} \times xb_{j}^{t} + \varepsilon_{2} \times x_{ij}^{t} |)/K$$

In Eq. (18), $x_{ij}^{t + 1}$ denotes the position $x_{ij}^{t}$ of the *i*th flamingo in the *j*th dimension of the population in the (*t* + 1)th iteration, *x* denotes the position of the *i*th flamingo in the *j*th dimension of the flamingo population in the *t*th iteration, i.e., the position of the flamingo foot. *xb*_*j*_^*t*^ denotes the *j*th dimensional position in the population of the best adapted flamingo in *t* iterations. *k* = *K* (*n*) is the diffusion factor, which is a random number with a cardinal distribution of *n* degrees of freedom. It is used to increase the size of the flamingo's foraging range and to simulate the chances of individual selection in nature, it is increasing global meritocracy.$G_{1} = N(0,1)$ and $G_{2} = N(0,1)$ are random numbers that follow a standard normal distribution,$\varepsilon_{1}$ and $\varepsilon_{2}$ are randomized by − 1 or 1.

### Migration behavior

When food is scarce in the current foraging area, the flamingo population migrates to the next more abundant food area. Assuming that the location of the food rich area in dimension *j*th is *xb*_*j*_, the migration equation for the flamingo population is as follows.

19 $$x_{ij}^{t + 1} = x_{ij}^{t} + \omega \times (xb_{j}^{t} - x_{ij}^{t} )$$

In Eq. (19), *x*_*ij*_^*t*+1^denotes the position of the *i*th flamingo in the *j*th dimension of the population in *t* + 1 iterations, x^*t*^_*ij*_ denotes the position of the *i*th flamingo in the *j*th dimension of the flamingo population in *t* iterations, i.e., the position of the flamingo foot. $xb_{j}^{t}$ denotes the *j*th dimensional position of the adapted flamingo in the population in *t* iterations. $\omega = N(0,N)$ is a random number with *N* degrees of freedom, it is used to increase the search space during the migration of flamingos, and it is also used to simulate the randomness of individual behavior of flamingos during specific migration.

### The algorithmic flow of FSA

Step 1: Initialize the axis net total swing function population, with the population set to* P* and the maximum number of iterations to $Iter_{Max}$, and the proportion of migrating flamingos in the first part $MP_{b}$.

Step 2: The number of foraging flamingos in the *i*th iteration of the flamingo population update is $MP_{{\text{r}}} = rand[0,1] \times P \times (1 - MP_{b} )$. The number of migrating flamingos in the first part of this iteration is $MP_{0} = MP_{b} \times P$. The number of migrating flamingos in the second part of this iteration is $MP_{{\text{t}}} = P - MP_{0} - MP_{r}$, and the fitness values of individual flamingos are obtained, and the flamingo population is ranked according to the fitness values of the individuals. The $MP_{b}$ former flamingos with low fitness and the former flamingos $MP_{{\text{t}}}$ with high fitness are considered migratory flamingos, while the others are considered foraging flamingos.

Step 3: Update migrating flamingos according to Eq. (19) and foraging flamingos according to Eq. (18).

Step 4: Check for out-of-bounds flamingos.

Step 5: Reach the maximum number of iterations and go to Step6; otherwise, execute Step2.

**Figure 5:**
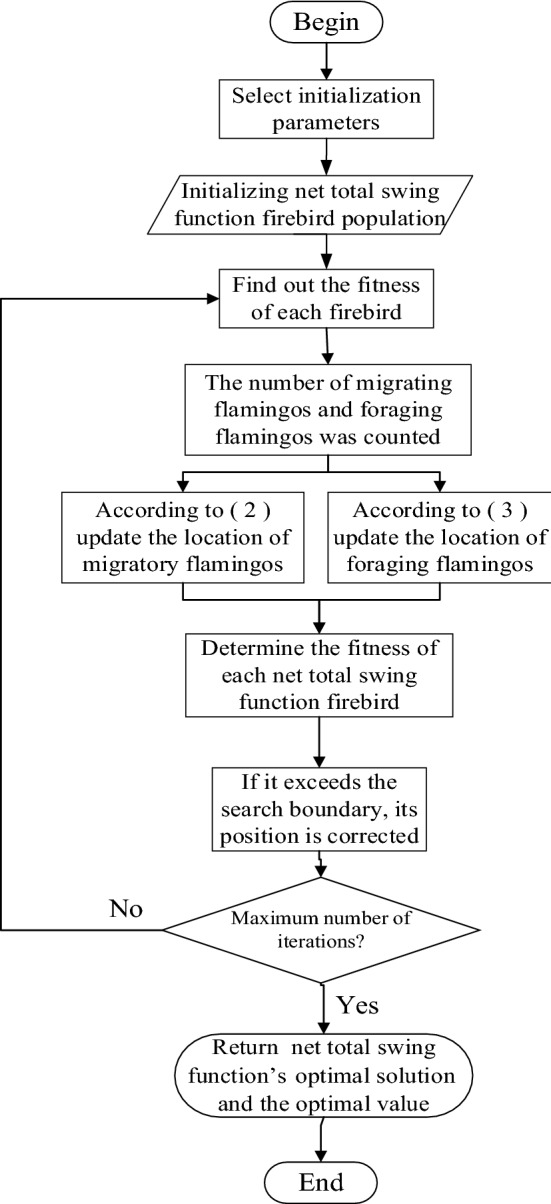
Flow chart of FSA algorithm.

Step 6: Output axis net total swing function’s the optimal solution and the optimal value. The flow chart of FSA is shown in Fig. 5.

## Results and discussion

### Datasets

This paper is based on the maintenance data of a hydraulic power maintenance company from 2015 to the 2020 year. Due to the small number of maintenance visits of the disassembled generator set every year, there are 200 pieces of maintenance data in this data set, among which each piece of data has 4 kinds of characteristic data, and it is including absolute swing, relative swing, net total swing and maximum net total swing. Because this type of unit is commonly used by hydro-electric power maintenance companies, this data set has become an important reference data set. In this paper, a prediction study is conducted on the 1–80 times data of the maintenance data set, which consists of eight measuring points in a circle of the large axis and a total of 2400 items. Among them, there are 640 pieces of net total swing data. Maintenance workers mainly use the net total swing as a reference standard to carry out work. Therefore, net total swing and maximum net total swing are screened separately as data sets. The data of the first 70 items are the training set, and the data of the last 10 items are the test set. 71–80 times of data are used for research in this paper. Table 1 is the data of the net total pendulum.

**Table 1:** Net total swing of measurement points (71–80 times).

Serial number	Measurement point I (mm)	Measurement point II (mm)	Measurement point III (mm)	Measurement Point IV (mm)	Measurement Point V (mm)	Measurement point VI (mm)	Measurement point VII (mm)	Measurement point VIII (mm)
1	25	37	49	8	−25	−37	−49	−8
2	14	−4	−20	−26	−14	4	20	26
3	12	−4	−20	−24	−12	4	20	24
4	16	20	25	3	−16	−20	−25	−3
5	19	23	14	−2	−19	−23	−14	2
6	14	21	16	−3	−14	−21	−16	3
7	16	20	16	−4	−16	−20	−16	4
8	14	−7	−24	−26	−14	7	24	26
9	−17	−19	−11	7	17	19	11	−7
10	6	3	−2	−4	−6	−3	2	4

The data set is obtained by using the Marr scale and measurement software. The data measured by the Marr scale is an absolute pendulum, and then the net total pendulum, relative total pendulum, and maximum net total pendulum are obtained by a series of axis adjustment formulas.

To verify the accuracy and validity of the improved GM(1, 1) model established in this study, historical data at the suspended second guide flange are selected and predictions are obtained 1–80 times, and GM(1, 1), BP, SVM, and PINN are selected for comparison.

**Figure 6:**
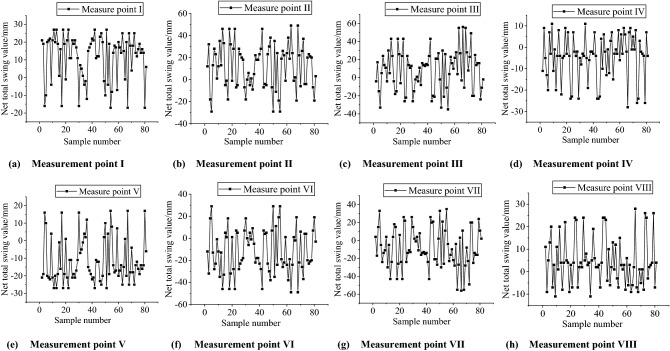
Trend of net full swing at eight measurement points on the axis.

The pendulum trend of the net total swing of the axis of the eight measurement points can be observed in Fig. 6, from which it can be found that the data of each measurement point shows oscillatory nature, it is not a single increasing or decreasing trend, so the traditional GM(1, 1) is not very applicable to it, the oscillation trend needs to be weakened.

**Figure 7:**
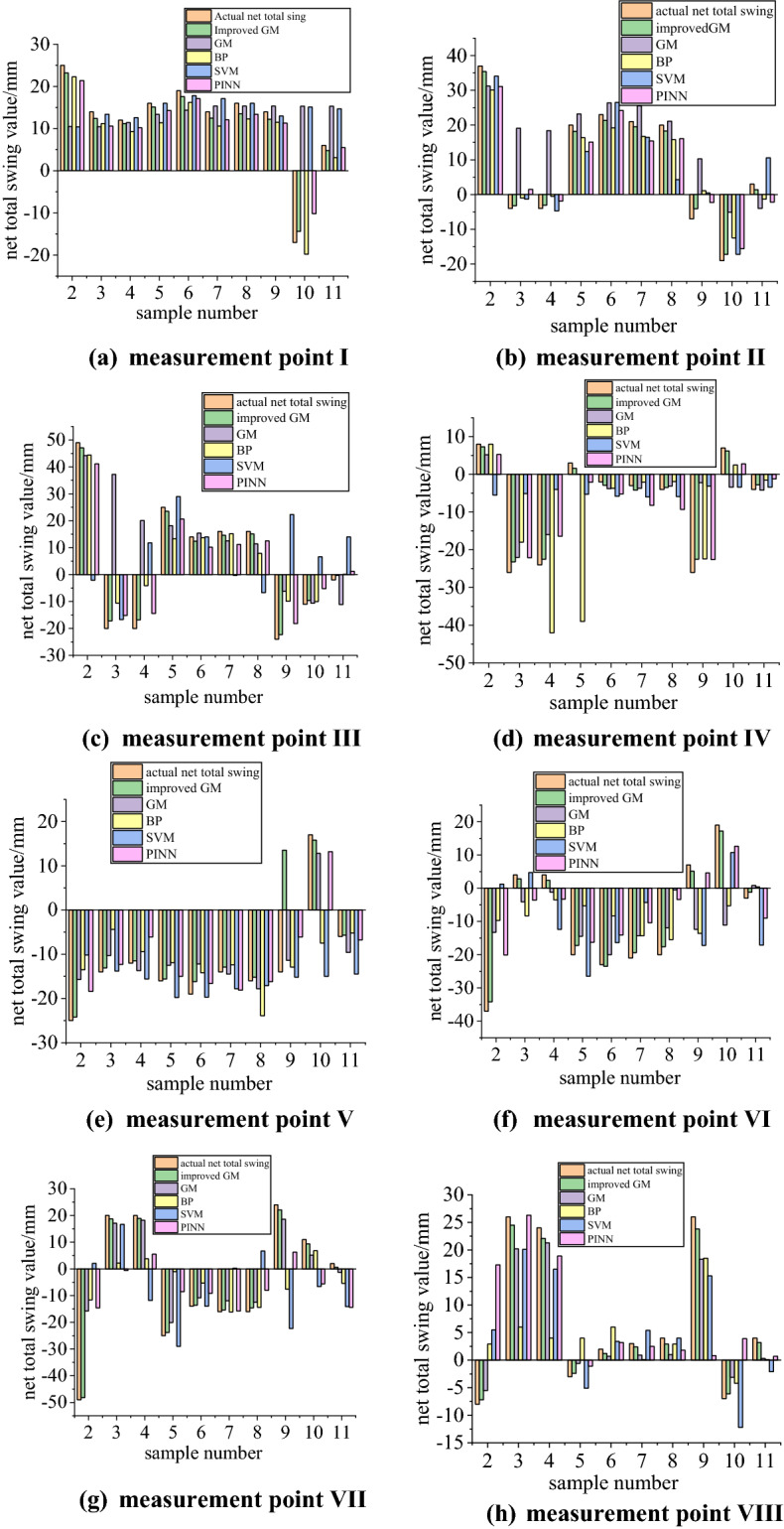
Comparison of predictions by the model.

In this experiment, the axis net total swing sequence with oscillating wave phenomenon is processed by accelerating translation and meaning transformation method to weaken the net total swing oscillation sequence. GM(1, 1) is improved by using this method, and the net total swing is taken as a predictive variable. At the same time, GM(1, 1), BP, SVM, and PINN models are selected for comparison, and the net total swing of 71–80 times axis is predicted respectively, with the first 70 times as the training value and 71–80 times as the predicted value. The actual value; predicted value, and relative error of net total swing prediction is shown in Fig. 7.

**Figure 8:**
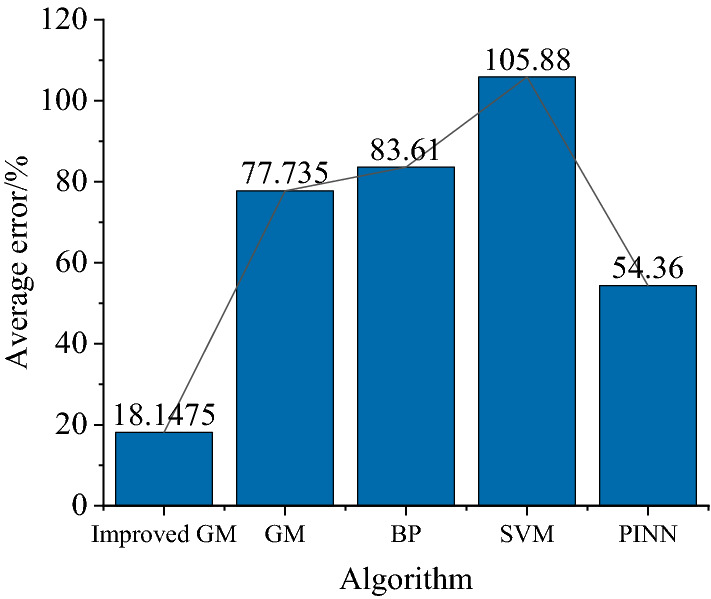
Comparison of average errors.

It can be seen from Fig. 6 that the prediction model of net total swing based on the improved GM(1, 1) axis proposed in this paper is closer to the actual net total swing of the measurement point than the predicted value of other algorithms. To accurately analyze the prediction accuracy of several models, the error comparison analysis of the improved GM(1, 1), GM(1, 1), BP, SVM, and PINN is carried out. In addition, Due to the randomness of the neural network, each structural model has to go through ten times training and verification during parameter setting. The errors obtained are shown in Table 2, and the comparison of the average errors of the eight measurement points is shown in Fig. 8.

**Table 2:** Error analysis of data predicted by different models.

Algorithm	Measurement point I (%)	Measurement point II (%)	Measurement point III (%)	Measurement point IV (%)	Measurement point V (%)	Measurement point VI (%)	Measurement point VII (%)	Measurement point VIII (%)	Average error (%)
Improved GM	11.27	18.3	17.04	22.48	25.23	20.99	12.62	17.25	18.1475
GM	49.69	176.45	111.69	56.13	25.35	107.17	42.14	53.26	77.735
BP	22.68	55.04	41.32	181.39	41.32	129.61	95.49	102.03	83.61
SVM	43.71	61.59	169.24	119.82	49.05	162.83	169.74	71.06	105.88
PINN	18.1	52.01	39.13	70.21	23.11	70.11	110.12	52.16	53.36

As can be seen from Fig. 8, compare with traditional GM(1, 1), BP, SVM, and PINN, after weakening the axis net total swing with oscillations combined with accelerated translation and mean transformation, the average error of GM(1, 1) model established by sum factor transformation is smaller than that of other models, it is indicating high prediction accuracy. The problem of the large prediction error of other algorithms for oscillating wave sequence is solved.

This study also compares three loss functions such as mean absolute error (MAE), root mean square error (RMSE) and mean absolute percentage error (MAPE), as shown in Table 3.

**Table 3:** Loss function comparison diagram.

	MAE	RMSE	MAPE (%)
Improved GM	1.78	0.134	2.1
GM	6.59	10.72	17.4
BP	8.30	10.63	34
SVM	11.69	14.52	4.1
PINN	5.32	8.23	3.3

In this study, the predicted values of 71–81 axis net full swing of five algorithms are compared as loss functions. From Table 3, it is found that the MAPE between the improved GM, SVM and PINN are relatively close to 2.1%, 4.1% and 3.3% respectively. Compared with GM, BP and SVM, PINN has smaller MAE, RMSE and MAPE, and there is still a certain gap compared with the improved GM. It can be concluded that the improved GM is more suitable for the data prediction of axis net full swing.

**Figure 9:**
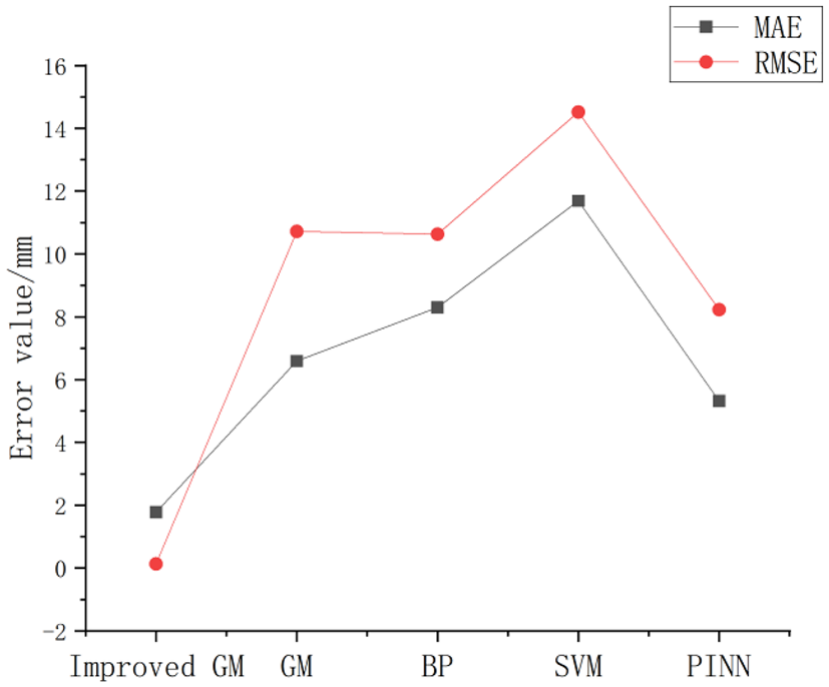
Comparison of algorithm MAE and RMSE.

**Figure 10:**
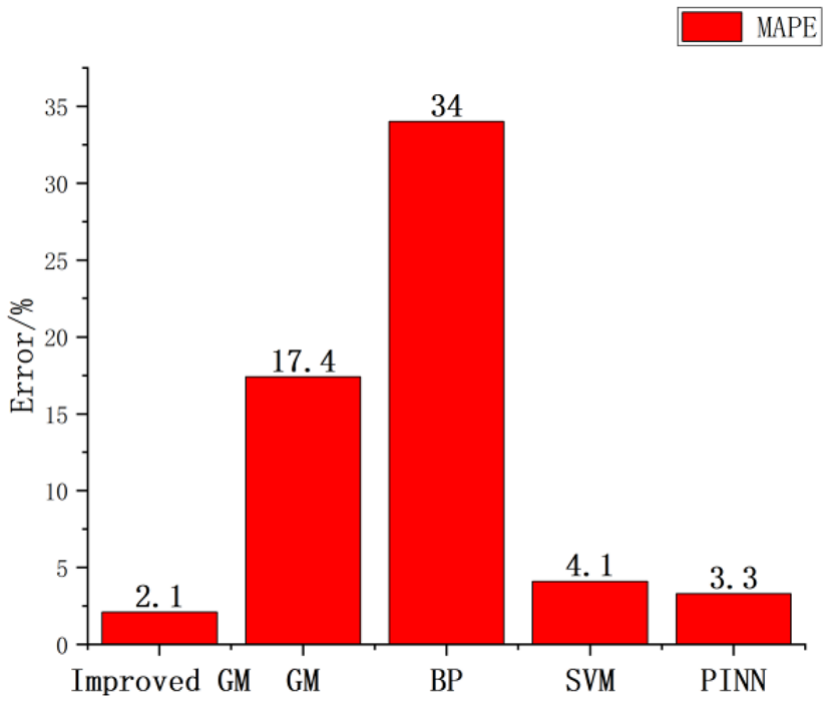
Comparison of algorithm MAE and RMSE.

It is more obvious from Figs. 9 and 10 that the average MAE and RMSE of the improved GM are the smallest, the PINN is a little worse, and the GM, BP and SVM are much worse. The improved GM is better for the data such as the net full swing of the hydraulic turbine generator unit, because it is closer to the real data, with less error, and has more reference value for the maintenance personnel of the hydraulic turbine generator unit. It can make better judgments on the axis abnormalities and improve the axis adjustment efficiency.

The sine function curve of axes net total swing is constructed by the improved GM(1, 1) eight-point net total swing prediction. The axial net total swing sine functions for the 71st-80th are shown in Table 4.

**Table 4:** Sine function of net full swing for 71–80 sub-axes.

Number of times	The net total swing sine function
71	−0.03513 + 22.09*cos(x*0.01756) + 40.96*sin(x*0.01756)
72	0.3865 + 14.04*cos(x*0.01716) − 21.26*sin(x*0.01716)
73	0.293 + 12.47*cos(x*0.01721) − 20.37*sin(x*0.01721)
74	0.02027 + 14.22*cos(x*0.01739) + 20.43*sin(x*0.01739)
75	0.1227 + 18.76*cos(x*0.01727) + 13.77*sin(x*0.01727)
76	−0.2298 + 14.38*cos(x*0.01792) + 15.78*sin(x*0.01792)
77	−0.1641 + 15.83*cos(x*0.01774) + 14.59*sin(x*0.01774)
78	−0.1342 + 14.02*cos(x*0.01756) − 23.46*sin(x*0.01756)
79	0.2045—17.19*cos(x*0.01774) − 10.7*sin(x*0.01774)
80	0.09773 + 5.457*cos(x*0.01716) − 1.623*sin(x*0.01716)

The 71–80 times net total swing sine function is used as input of each method to search for the maximum value of net total swing sine function, and the maximum value obtained is the orientation adjustment. According to the results of 10 times of prediction, the obtained axis adjustment azimuth search results are shown in Table 5.

**Table 5:** Search results for axial adjustment orientation.

Number	Actual orientation (function maximum) (°)	Traditional handwork (°)	Error (°)	Particle group (math) (°)	Error (°)	Simulated annealing (°)	Error (°)	Flamingos (°)	Error (°)
71	60	45	15	61.287	1.287	61.28	1.28	60.69	0.69
72	321	315	6	308.626	12.37	310.82	10.18	319.62	1.38
73	289	315	26	305.735	16.73	308.1	19.1	288.45	0.55
74	59	45	14	55.361	3.639	55.115	3.885	57.46	1.54
75	40	45	5	36.664	3.336	37.665	2.335	38.29	1.71
76	48	45	3	46.416	1.584	46.324	1.676	46.56	1.44
77	67	90	23	41.976	25.02	49.624	17.37	65.44	1.56
78	294	315	21	299.034	5.034	296.08	2.08	293.33	0.67
79	223	215	8	208.475	14.52	210.69	12.31	221.48	1.52
80	340	315	25	349.825	9.825	349.03	9.03	340.69	0.69

**Figure 11:**
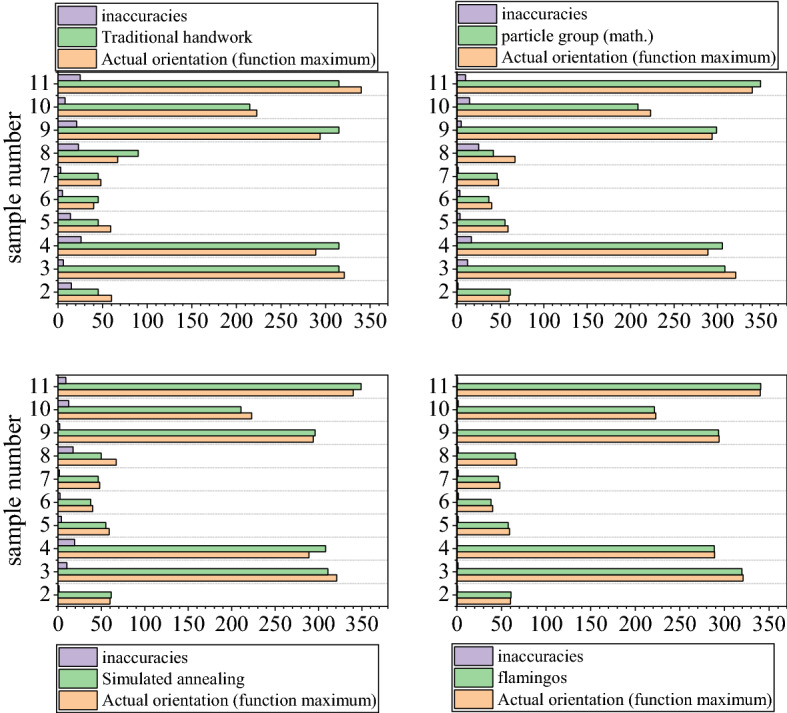
Comparison of search results by method.

As can be seen from Table 5, the search results of the simulated annealing algorithm are significantly smaller in error than the traditional manual and particle swarm methods, with high search accuracy and good stability. However, the search results that use the flamingo algorithm are close to the actual orientation and it has higher stability. In addition, the minimum error of the flamingo algorithm can reach 0.55, and the search results of each method compared with the actual orientation are shown in Fig. 11.

**Figure 12:**
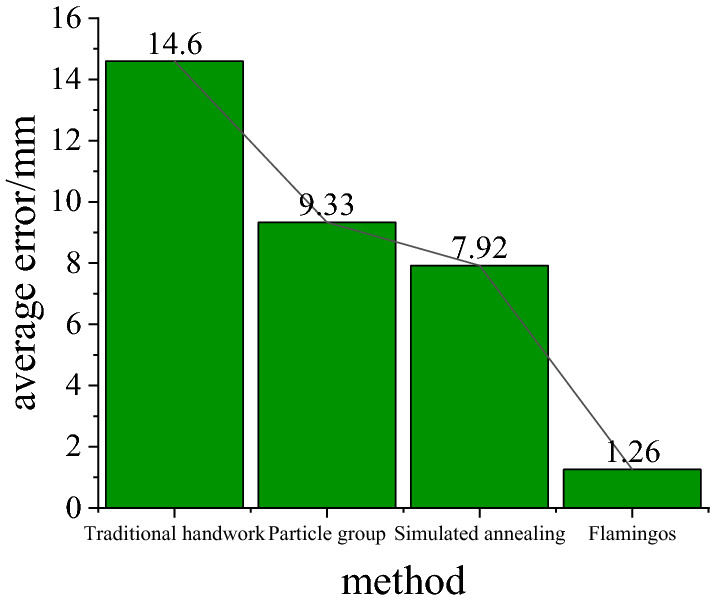
Comparison of the average error of each method.

In order to accurately analyze the accuracy of the axial adjustment orientation search of the centralized method, an error comparison is performed as shown in Table 6, as shown in Fig. 12. From the table, it can be seen that from the selected 10 experimental results, the maximum error of traditional manual is 26, the minimum error is 3, and the average error is 14.6, the maximum error of particle swarm is 25.024, the minimum error is 1.287, and the average error is 9.33, the maximum error of simulated annealing is 19.1, the minimum error is 1.28, and the average error is 7.92, and the maximum error of flamingo is the maximum error of flamingo is 1.71, the minimum error is 0.55, and the average error is 1.26. After the flamingo search algorithm seeks better than traditional manual by 13.4, better than particle swarm by 8.07, and better than simulated annealing by 6.66. The flamingo search algorithm is more significant in the Net total swing of axis seeking.

**Table 6:** Average error comparison table.

Algorithm	Traditional handwork/mm		Particle group (math.)/mm		Simulated annealing/mm		Flamingos/mm	
The quantity used as a parameter	Max	Min	Max	Min	Max	Min	Max	Min
Numerical value	26	3	25.024	1.287	19.1	1.28	1.71	0.55
Average error	14.6		9.33		7.92		1.26

## Conclusion

Axis adjustment method based on machine learning is an effective axis adjustment method. In this paper, our goal is used machine learning algorithm to improve the overall axis adjustment efficiency and axis adjustment accuracy. The main work is as follows: (I) A prediction model based on GM(1, 1) is proposed. The model includes accelerated translation and mean value transformation to pre-process the oscillatory axis net total swing series, so as to weaken the fluctuation of the axis net total swing series. (II) Determine the orientation of axis adjustment, in this paper, an optimization model of axis adjustment orientation based on flamingo search algorithm is proposed, the experimental results show that the average error of the flamingo search algorithm is 1.26, which can save more than 80% of the time compared with manual axis orientation search. It shows that the flamingo search algorithm-based axis orientation adjustment proposed in this paper is practical. The original traditional labor consumption affects the power generation in an average of 5 days. According to the average power generation of 62,000 kW units: 62,000 × 24 h × 5 days = 7.44 million kWh/unit, the method proposed in this paper can be shortened to 1 day, and it impact on power generation is only fifth of the previous one, that is, 1.488 million kWh/unit. Compared with the traditional manual stop axis maintenance operation, the axis measurement time in this paper can be reduced by more than 80%, the labor costs of each unit is calculated from the traditional 16 people, 5 days construction period, and 550 yuan per person: 5 days × 16 people × 550 yuan/day = 44,000 yuan/unit, which is reduced to 11,100 yuan/unit for 2 people per day. It provides a reliable basis for finding the axis deviation and twists of the unit in time and finding solutions, it improves directly and indirectly, economic benefits, and it has wide application prospects and it is suitable for axis adjustment. It provides a strong guarantee for the safe and stable operation of the power grid and a solid technical support for the maintenance of hydro-generator unit.

## Data Availability

The datasets generated and/or analysed during the current study are not publicly available due at the request of the hydropower company, the data used in this paper can not be made public because of the operation data of the hydropower company's own power equipment but are available from the corresponding author on reasonable request.
